# Quadrature 1D–LiDAR Sensing for Indirect Height Measurement of a Helical Bogie Spring

**DOI:** 10.3390/s26196183

**Published:** 2026-09-29

**Authors:** Claudio Floridia, Francisco Frade, Orlando Frazão

**Affiliations:** 1 INESC TEC—Institute for Systems and Computer Engineering, Technology and Science, 4169-007 Porto, Portugal; claudio.floridia@inesctec.pt (C.F.); up202205095@up.pt (F.F.); 2 Faculty of Sciences, University of Porto, 4169-007 Porto, Portugal

**Keywords:** LiDAR, quadrature, phase analysis, condition monitoring, helical bogie spring

## Abstract

Three-dimensional optical ranging has become an effective tool for railway infrastructure monitoring, enabling accurate geometric characterization of tracks and the detection of structural anomalies. Beyond track inspection, this work investigates the potential of a low-cost optical ranging approach for indirect monitoring of railway suspension components. Two low-cost 1D laser ranging sensors arranged in quadrature were used to observe the lateral profile of a helical bogie spring under loading conditions. Owing to the 18° field of view of each sensor, the measured responses exhibited a quasi-sinusoidal dependence on spring height. By applying phase unwrapping to the quadrature signals, the spring height was reconstructed in real time. Two independent vertical displacement tests were performed, achieving mean errors of 0.66 mm and −1.47 mm, with standard deviations of 2.35 mm and 2.16 mm, respectively, demonstrating consistent accuracy and repeatability of the method. The proposed quadrature sensing approach provides a simple, non-contact, and cost-effective solution for indirect estimation of the bogie spring height. It is particularly suitable for installations where direct vertical measurements are impractical and offers a promising method for monitoring spring compression and suspension loading in railway condition monitoring systems.

## 1. Introduction

In railway engineering, suspension systems play a critical role in ensuring ride comfort, stability, and operational safety, particularly given increasing demands for higher operating speeds and reduced maintenance costs [1]. It is widely recognized that incorrect loading practices and poor cargo distribution across railway wagons are among the leading causes of suspension system failures, contributing to derailment risk, as discussed in [2]. Suspension components, including coil springs, are subjected to varying loads during railway operation, resulting in changes in their deformation and displacement. For Y25 bogies, coil springs are an integral part of the primary suspension system, and their mechanical response varies with the applied load [3].

Non-contact optical techniques have been extensively employed for displacement and deformation measurements owing to their high spatial resolution and accuracy. Among the most established approaches are fringe projection, interferometry, and digital image correlation (DIC) [4,5,6]. A comprehensive review of optical methods for non-contact vibration, continuous deformation, and moving-object measurements is presented in [7].

Active optical measurement techniques constitute an important class of non-contact methods for three-dimensional (3D) shape, displacement, and deformation measurement. Following a broad classification according to the modulation principle used to encode geometric information, optical 3D measurement methods can be divided into temporally modulated and spatially modulated approaches. Temporally modulated methods include time-of-flight (ToF) techniques, such as pulsed ToF LiDAR and continuous-wave or indirect ToF systems, in which range information is obtained from the temporal delay or phase relationship between the transmitted and received optical signals [8,9]. ToF LiDAR technology, based on both direct and indirect ToF determination [8], has emerged as a versatile sensing solution in numerous applications, including unmanned aerial vehicles (UAVs) [10].

Spatially modulated structured-light methods [11] encode surface geometry through the spatial variation of projected patterns and include established approaches such as fringe projection, Fourier transform profilometry [12], phase-shifting profilometry [13], Moiré-based techniques [14,15,16], modulation measuring profilometry [17,18], and phase-differencing profilometry [19]. These methods can provide high spatial resolution and accurate full-field 3D reconstruction, and recent developments have addressed high-speed acquisition, single-shot operation, phase unwrapping, and data-driven phase estimation [19,20,21,22,23].

However, many structured-light implementations rely on projector–camera configurations and, particularly in phase-shifting approaches, on multiple spatially encoded patterns. Such requirements can increase system complexity and may constrain their application in compact, low-cost, or real-time monitoring systems.

In railway applications, a method for estimating the wheel-rail contact force by measuring wheel-web deflection using three high-precision laser distance sensors mounted on the bearing box was proposed in [24]. More recently, a binocular-camera system based on structured light and density-based clustering was demonstrated for measuring the geometric parameters of bogie steel springs [25]. ToF LiDAR has been applied to infrastructure inspection, particularly railway track monitoring [26], while fixed LiDAR systems have also been reported for obstacle detection on railway tracks [27].

In parallel, optical fiber sensing technologies have also been investigated for structural health monitoring in railway systems, particularly for bogie condition assessment. Fiber Bragg grating (FBG) sensors enable simultaneous measurements of strain, vibration, and temperature with high sensitivity and immunity to electromagnetic interference [28], whereas distributed optical fiber sensing based on Brillouin scattering enables real-time strain monitoring and the detection of localized structural anomalies [29]. Although these techniques provide valuable monitoring capabilities, they generally require dedicated instrumentation, careful calibration, and relatively complex signal processing. Consequently, there is continued interest in simpler non-contact sensing approaches capable of extracting structural information from reduced measurement configurations.

Despite these advances, a scientific gap remains in the development of simple signal-processing approaches capable of reconstructing the displacement of helical components using reduced measurement configurations. For spring-like structures, the helical geometry naturally produces a periodic response along the axial direction. Under suitable conditions, the measured signal can therefore be described by sinusoidal or quasi-sinusoidal functions whose phase is directly related to the spatial position. Consequently, phase retrieval provides a natural framework for converting oscillatory measurements into continuous displacement estimates.

This work investigates the feasibility of using a pair of low-cost ToF 1D–LiDAR sensors to indirectly monitor the vertical displacement of a railway bogie spring by measuring two horizontal distances. Unlike conventional displacement measurement approaches, the proposed method reconstructs the spring displacement from the phase of two periodic signals acquired in quadrature, rather than directly from the measured distances.

As the LiDAR fields of view interrogate the lateral profile of the helical spring, the measured distance varies periodically with spring height because successive coils intersect different regions of the sensor field of view (FoV). By positioning the sensors in quadrature, these quasi-sinusoidal responses form an in-phase and quadrature (I–Q) signal pair, enabling continuous phase tracking and indirect reconstruction of the spring displacement through phase unwrapping.

Experimental validation demonstrated accurate displacement reconstruction, with mean errors of 0.66 mm and −1.47 mm and standard deviations of 2.35 mm and 2.16 mm for two independent vertical displacement tests. The proposed quadrature sensing technique provides a simple, low-cost, and non-contact solution for indirect spring displacement measurement, making it particularly suitable for railway condition-monitoring applications where direct vertical measurements are impractical. Although demonstrated using low-cost LiDAR sensors with a relatively wide field of view, the proposed methodology is not restricted to this technology. The underlying principle relies on the availability of a sufficiently periodic measurement response from the target geometry and can therefore potentially be extended to other displacement sensing technologies, including ultrasonic, capacitive, laser triangulation, and linear encoder sensors, provided that their outputs exhibit sufficient periodicity and stability for reliable phase retrieval.

## 2. Principle of Operation

**Figure 1:**
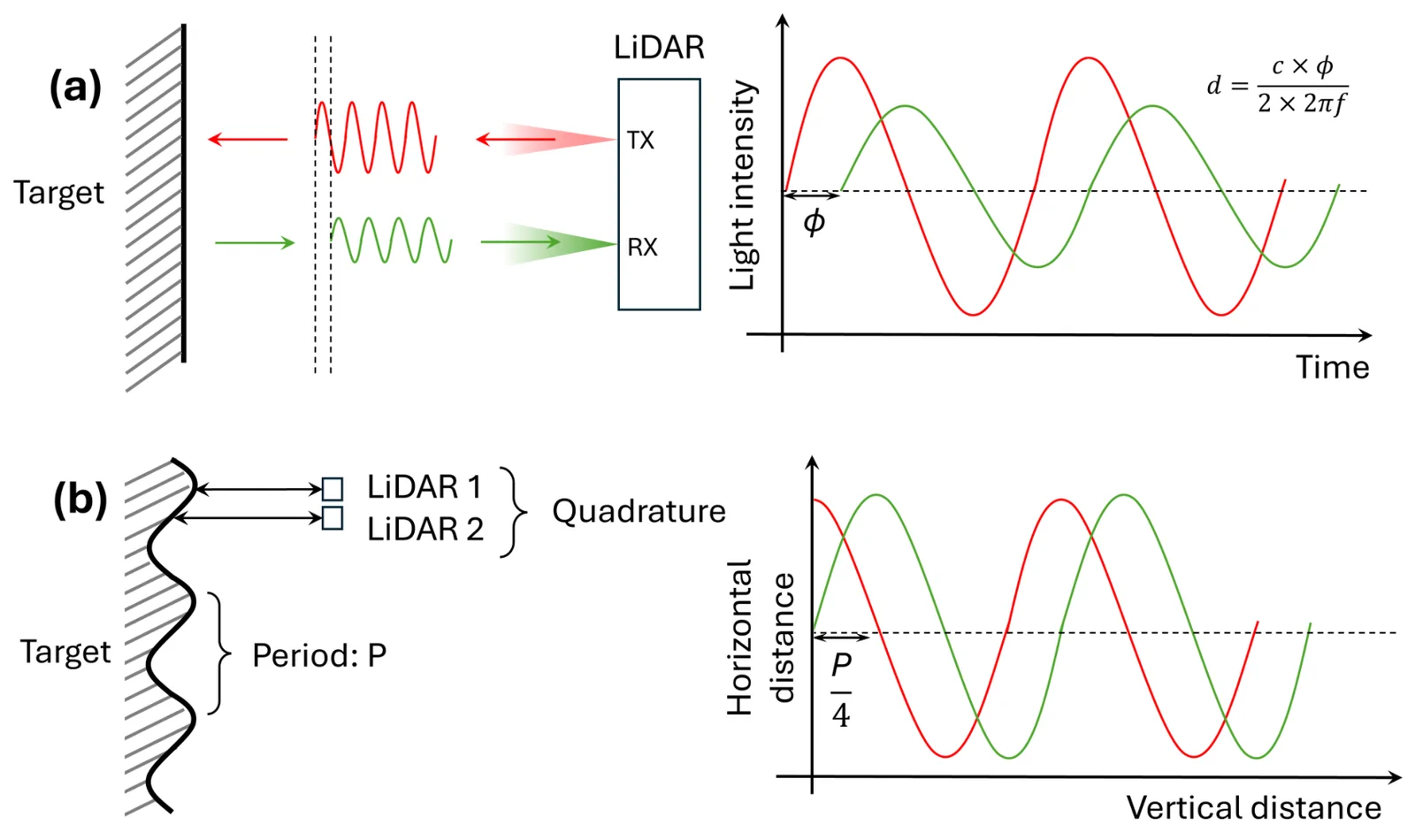
Principle of operation: (**a**) phase-based ranging in an iToF LiDAR; (**b**) two 1D ToF LiDARs in quadrature observing a periodic target.

The proposed method is conceptually analogous to phase-based ranging techniques used in some ToF LiDAR systems  [8], but the phase exploited here does not arise from optical propagation delay. Instead, it originates from the periodic spatial geometry of the helical spring. Specifically, in indirect time-of-flight (iToF) LiDAR systems, distance can be determined from the phase shift between the emitted and received modulated optical signals, as depicted in Figure 1a. The inset shows the expression used to determine the target distance. In this expression, *d* denotes the LiDAR-to-target distance, *c* the speed of light, *f* the amplitude-modulation frequency, and ϕ the phase shift between the emitted and reflected light.

In this work, a similar principle is adopted; however, rather than relying on signal propagation delays, the method exploits the periodic geometry of the target. Two 1D LiDAR sensors are arranged in quadrature such that their horizontal distance measurements exhibit a phase difference of one quarter of the structural period, producing sinusoidal and cosinusoidal responses, as in Figure 1b. The vertical displacement of the LiDAR pair can then be inferred from these horizontal measurements by evaluating and tracking the phase of two in-phase and quadrature signals, $S_{1}$ and $S_{2}$, as given by Equation (1):

(1) $$\begin{array}{cc} S_{1} & =A\cos(\frac{2\pi }{P}h+\theta_{0})+B\\ S_{2} & =A\sin(\frac{2\pi }{P}h+\theta_{0})+B \end{array}$$

where *A* is the amplitude, *B* is the offset, *P* is the structural period (pitch), $\theta_{0}$ is the initial phase.

The height, *h*, is therefore estimated from the recovered and unwrapped phase using Equation (2):

(2) $$h=\frac{\theta_{\mathrm{unwrap}}-\theta_{0}}{2\pi /P}$$

where $\theta_{\mathrm{unwrap}}$ is the retrieved unwrapped phase.

## 3. Experimental Setup

For the experimental setup, a bogie spring was used with the characteristics presented in Table 1.

**Figure 2:**
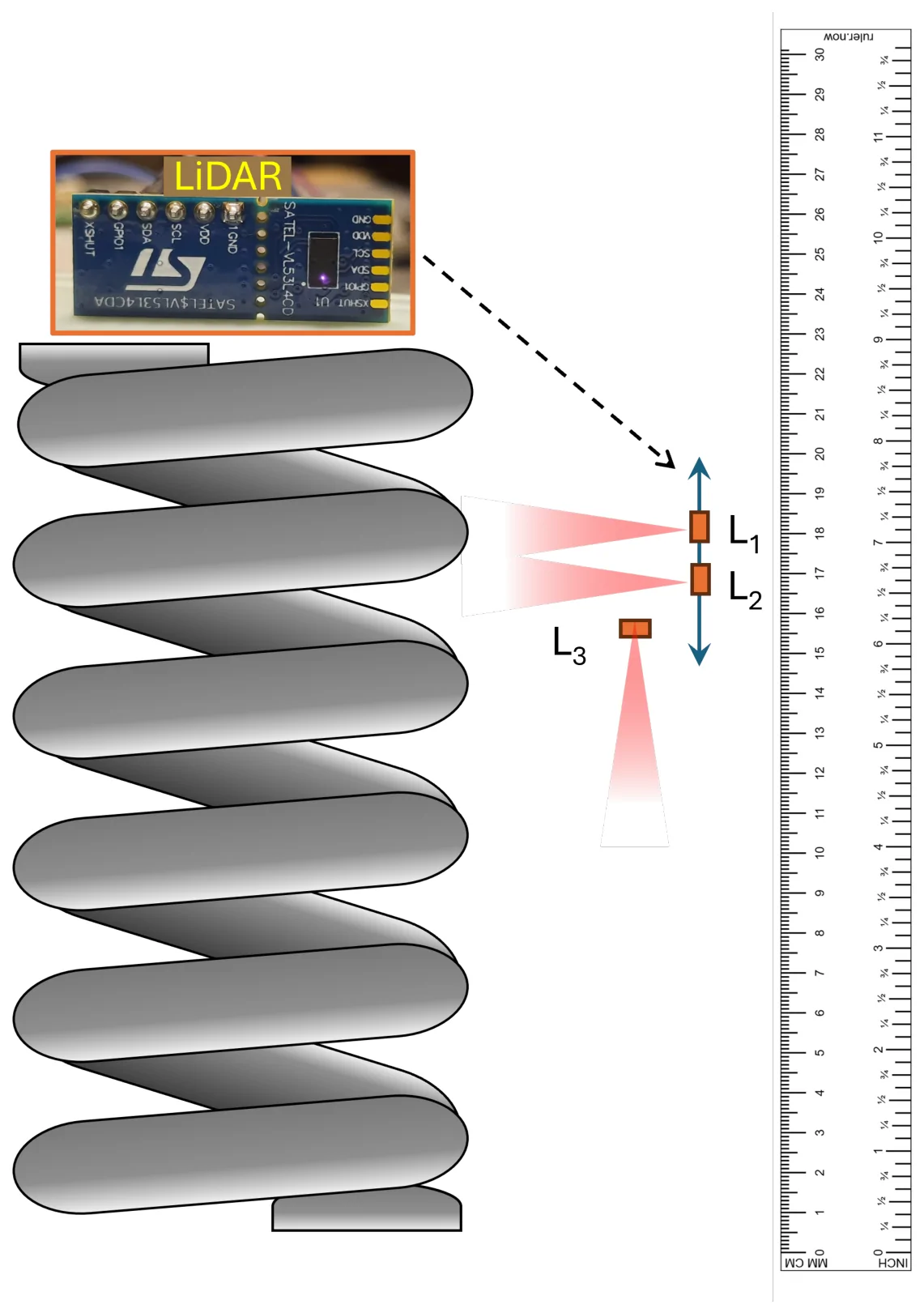
Experimental setup scheme enabling variation in the LiDAR height while measuring the horizontal distance *x*, in millimeters. The inset shows a close-up view of one of the VL53L4CD sensors.

The setup consisted of positioning the LiDARs in the configuration illustrated in Figure 2, at a convenient distance from the spring. Two LiDAR sensors were placed facing the spring, while a third LiDAR was oriented downward to measure the height, which was also verified using a ruler. In the first set of experiments, a single horizontal LiDAR was used to characterize the spring profile response as a function of the height, while for real-time characterization and height evaluation, the two LiDARs, $L_{1}$ and $L_{2}$, were placed in quadrature with respect to each other, with the quadrature separation defined as $\frac{1}{4}\;P$, where *P* is the pitch between coils listed in Table 1, and the third LiDAR, $L_{3}$, was used to record the height. All three sensors, $L_{1}$, $L_{2}$, and $L_{3}$, are time-of-flight LiDAR sensors, model VL53L4CD. They have a measurement range from 1 mm to 1200 mm, a resolution of 1 mm, a field of view (FoV) of 18°, and an operating wavelength of 940 nm. The devices are controlled by an Arduino Uno R3, based on the ATmega328 microcontroller, with 32 KB of flash memory and interfaces including I2C, SPI, UART, and USB.

## 4. Experimental Results

Using the proposed experimental setup, two experimental approaches were carried out. First, a single LiDAR was used to characterize the coil-distance response as a function of height, with measurements taken millimeter by millimeter using a ruler. For the single-LiDAR approach, the measured responses were fitted, and the corresponding quadrature component was synthesized.

Second, three LiDAR sensors were used simultaneously to characterize and track the indirectly estimated height. For the three-LiDAR approach, two sensors were arranged in physical quadrature, while the third sensor was used as a reference for the spring height.

### 4.1. Single LiDAR Characterization

**Figure 3:**
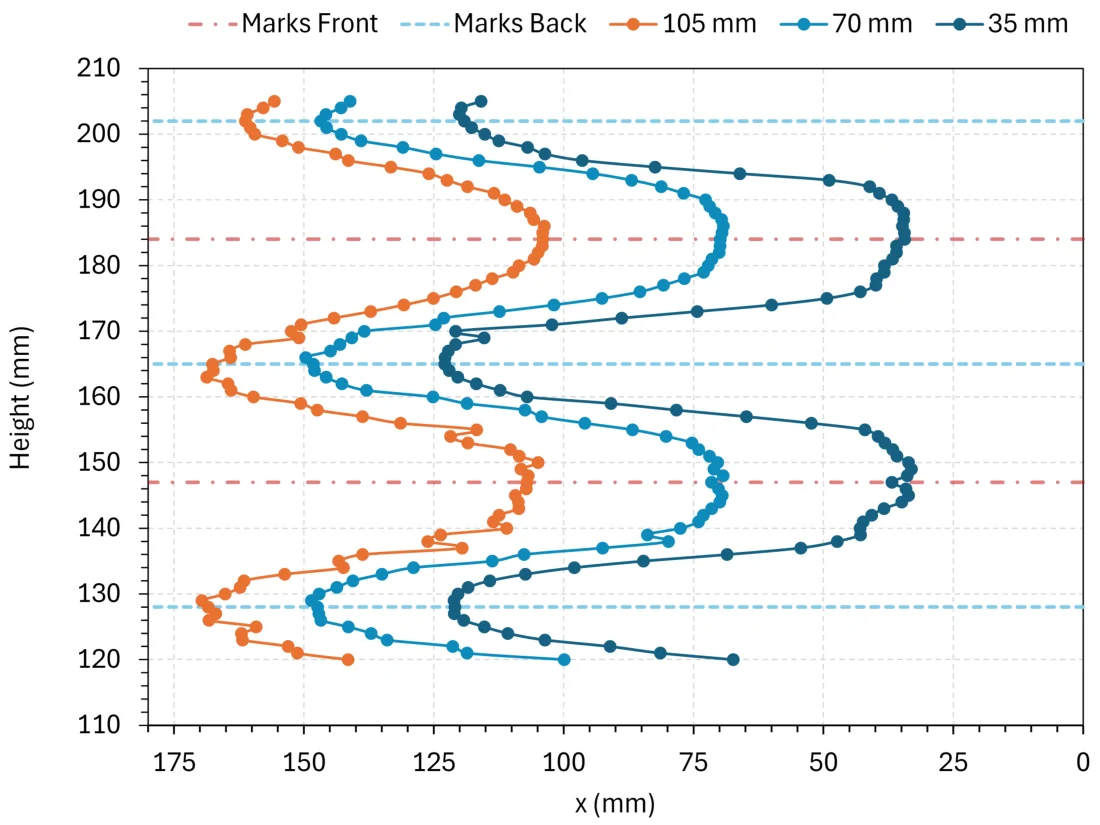
Height scans for three different spring-to-LiDAR distances. The desired sinusoidal behavior is observed at the largest distance. Height is shown on the vertical axis to facilitate comparison with the configuration in Figure 1.

In this characterization, the distance between the spring and the LiDAR, *x*, in millimeters was measured as a function of height relative to the base for three different spring positions: 35 mm, 70 mm, and 105 mm. For each measurement point, 50 samples were acquired and the average value was used in the plot. The results are shown in Figure 3. The measured responses were subsequently fitted to obtain the corresponding quadrature component, as described later in this section.

The height is represented on the vertical axis to facilitate comparison with the spring configuration shown in Figure 2. The results presented in Figure 3 show that the LiDAR response as a function of height exhibits a periodic behavior that becomes increasingly sinusoidal as the horizontal distance between the device and the spring increases. This behavior is consistent with the helical geometry of the spring, where the measured distance alternates between the inner and outer regions of the coil along the vertical scan. These scans enable the extraction of key geometric parameters of the spring. In particular, the signal periodicity is directly related to the spring pitch, yielding a value of 37 mm for measurements acquired from both the front and the back of the coil, as indicated by the dashed and dotted-dashed lines in Figure 3.

The signal amplitude reflects the radial dimensions of the structure, with values of 65.9 mm, 80.4 mm, and 89.8 mm for LiDAR distances of 105 mm, 70 mm, and 35 mm from the spring, respectively. The latter value is in good agreement with the expected value of 90 mm (114 mm − 24 mm) obtained from Table 1. In other words, placing the laser-based sensor closer to the spring provides a better estimate of the coil depth; however, sinusoidal behavior is lost. For larger distances, the measurement integrates the geometric variations more smoothly, resulting in a signal that more closely approximates a sinusoidal function. This characteristic enables the extraction of key geometric parameters of the spring. In particular, the periodicity of the signal is directly related to the spring pitch, while the amplitude reflects the radial dimensions of the structure when the LiDAR is placed close to the target, as expected from the FoV of this device. Furthermore, by applying a sinusoidal fitting and phase retrieval approach, it becomes possible to estimate the vertical position along the spring, allowing the reconstruction of its profile.

**Figure 4:**
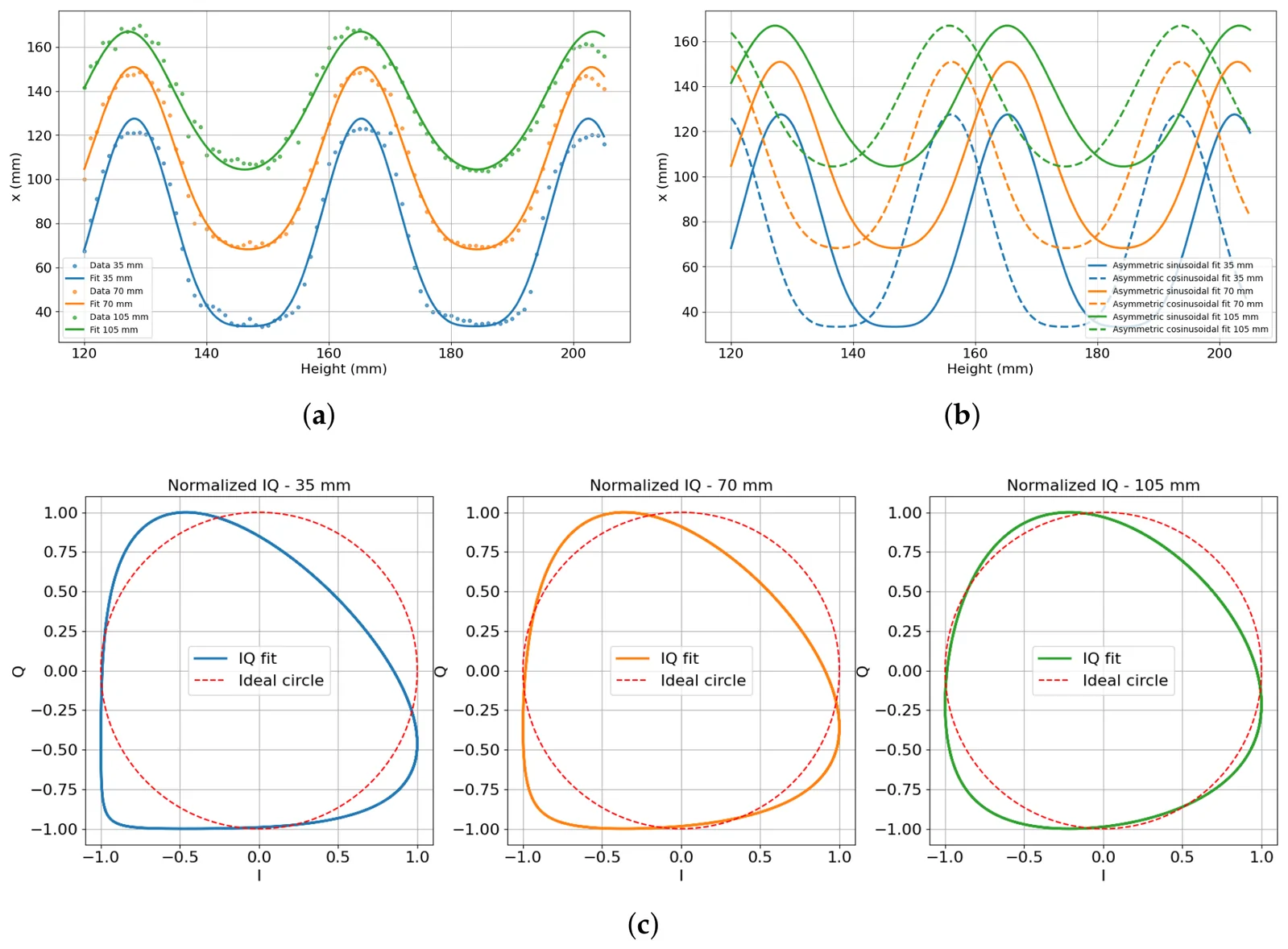
Sinusoidal expansion fits of the experimental data for the three different target distances: (**a**) Original data and their fits. (**b**) Sinusoidal and orthogonal expansions. (**c**) Normalized I–Q plots of fits compared with the ideal circle.

To characterize the LiDAR response, the experimental data shown in Figure 3 were fitted using the sinusoidal model given by Equation (3), where the sine function was chosen as the reference representation. An orthogonal component was then generated by replacing the sine term with a cosine term, yielding Equation (4). The resulting pair of signals forms an orthogonal (I/Q) representation, from which the phase can be estimated using the four-quadrant inverse tangent function.

(3) $$R=A\;\sin\left(\frac{2\pi }{P}h+\theta_{0}\right)\left[1-k\left(1-\sin(\frac{2\pi }{P}h+\theta_{0})\right)\right]+B$$

(4) $$O=A\;\cos\left(\frac{2\pi }{P}h+\theta_{0}\right)\left[1-k\left(1-\cos(\frac{2\pi }{P}h+\theta_{0})\right)\right]+B$$

where *R* and *O* are the reference and orthogonal components, respectively, both corresponding to the measured horizontal distance between the sensor and the spring coil, *h* is the LiDAR height, and *A*, *B*, *P*, $\theta_{0}$, and *k* are fitting parameters. Table 2 presents the fitting parameters for Equations (3) and (4) used to generate Figure 4a,b.

The parameter *k* controls the waveform asymmetry caused by the sensor FoV by progressively compressing its lower portion while enhancing its upper portion. As *k* increases from 0.176 to 0.316, the waveform becomes increasingly asymmetric, corresponding to shorter LiDAR-to-target distances. In the limit k=0, the expressions for *R* and *O* reduce to purely sinusoidal and quadrature sinusoidal responses, respectively. The two orthogonal signals are hereafter also referred to as the I and Q components.

The fits obtained using Equation (3) are shown in Figure 4a. The orthogonal component can be derived directly from Equation (3) by replacing the sine function with a cosine function as in Equation (4). The corresponding results are presented in Figure 4b. The combination of these two expressions produces the expected I–Q (in-phase and quadrature) trajectories, shown in Figure 4c, provided that both *R* and *O* are normalized to the range [−1,1] according to Equations (5) and (6):

(5) $$R_{\mathrm{norm}}=2\cdot \frac{R-R_{\min}}{R_{\max}-R_{\min}}-1$$

(6) $$O_{\mathrm{norm}}=2\cdot \frac{O-O_{\min}}{O_{\max}-O_{\min}}-1$$

where $R_{\max}$, $O_{\max}$, $R_{\min}$, and $O_{\min}$ are the maximum and minimum values of *R* and *O*, respectively.

As observed, the response becomes increasingly sinusoidal as the distance increases, and the corresponding I–Q trajectory approaches the ideal circular shape.

**Figure 5:**
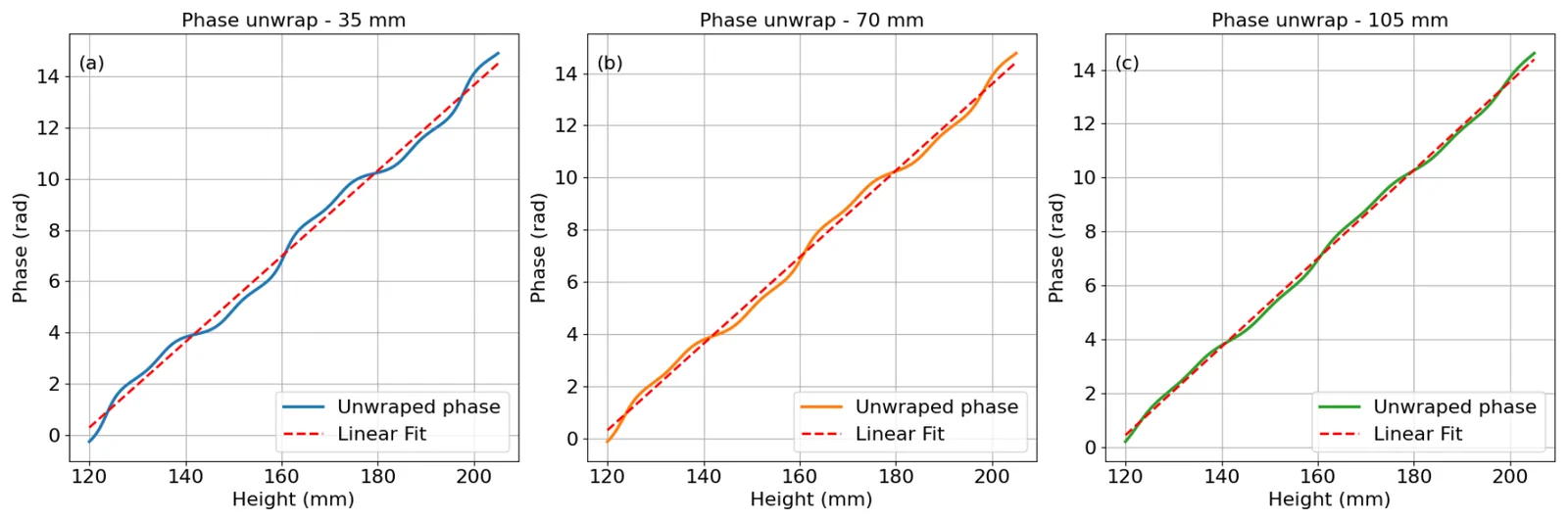
Unwrapped phase for the various spring-coil distances: (**a**) 35 mm, (**b**) 70 mm, and (**c**) 105 mm.

To understand the impact of the LiDAR position on the unwrapped phase response as a function of height, the results are plotted in Figure 5. The phase is calculated using Equation (3) and its cosine counterpart, Equation (4), according to Equation (7).

(7) θ=atan2(Q,I)

where θ is the phase, and atan2 is the modified arctangent function that returns the angle or phase in the range (−π,π]. By unwrapping the phase, the results shown in Figure 5 are obtained. As the sensor-to-spring distance increases from 35 mm to 105 mm, the response becomes more linear, as expected.

To quantify the proximity of the I–Q trajectory to an ideal unit circle and the unwrapped phase to an ideal linear response, the circle root-mean-square-error (RMSE) and phase RMSE were calculated for the different sensor-to-target distances using the smooth fitted signals of Equations (3) and (4). The circle RMSE was obtained by calculating the radial distance of each point in the fitted I–Q trajectory from the center of the I–Q plane and comparing it with the radius of the ideal unit circle. The RMSE was then computed as the square root of the mean squared radial deviations, providing a quantitative measure of the circularity of the fitted trajectory. Similarly, the phase RMSE was calculated as the square root of the mean squared deviations between the unwrapped phase and its linear fit, providing a quantitative measure of the deviation of the phase from linear behavior. The obtained values are reported in Table 3.

As the distance increases from 35 mm to 105 mm, both metrics decrease monotonically. The circle RMSE is reduced from 0.18 to 0.08, indicating that the I–Q trajectory progressively approaches the ideal unit-circle behavior. Similarly, the linear-phase RMSE decreases from 0.35 to 0.16, demonstrating that the unwrapped phase becomes increasingly linear with respect to height. These results quantitatively confirm the observations from Figure 4c and Figure 5 and justify the selection of the 105 mm configuration for the real-time height-tracking experiments, which are presented in the next section.

To experimentally validate these predictions, two LiDAR sensors, $L_{1}$ and $L_{2}$, were positioned in quadrature with respect to each other, while a third LiDAR sensor, $L_{3}$, was used to measure the height. The experimental setup and methodology are described in the following section.

### 4.2. Dual Quadrature LiDAR Measurement and Height-Tracking Algorithm

In this section, the results obtained using two 1D LiDARs placed in quadrature are presented. As indicated in Table 1, the pitch between coils is 37 mm, and thus the ideal quadrature distance between the devices is one-quarter of this distance, i.e., 9.25 mm. However, due to dimensional constraints, it is more convenient to place the LiDARs 46.25 mm apart (37 + 9.25 mm). In practice, the actual center-to-center separation between $L_{1}$ and $L_{2}$ was 45.72 mm, corresponding to a 0.53 mm deviation from the ideal quadrature position. Under the assumption of ideal sinusoidal and cosinusoidal responses, this positioning error introduces a corresponding phase offset in the reconstructed signal, consequently affecting the recovered height and resulting in an equivalent height deviation of the same magnitude as the positioning error.

**Figure 6:**
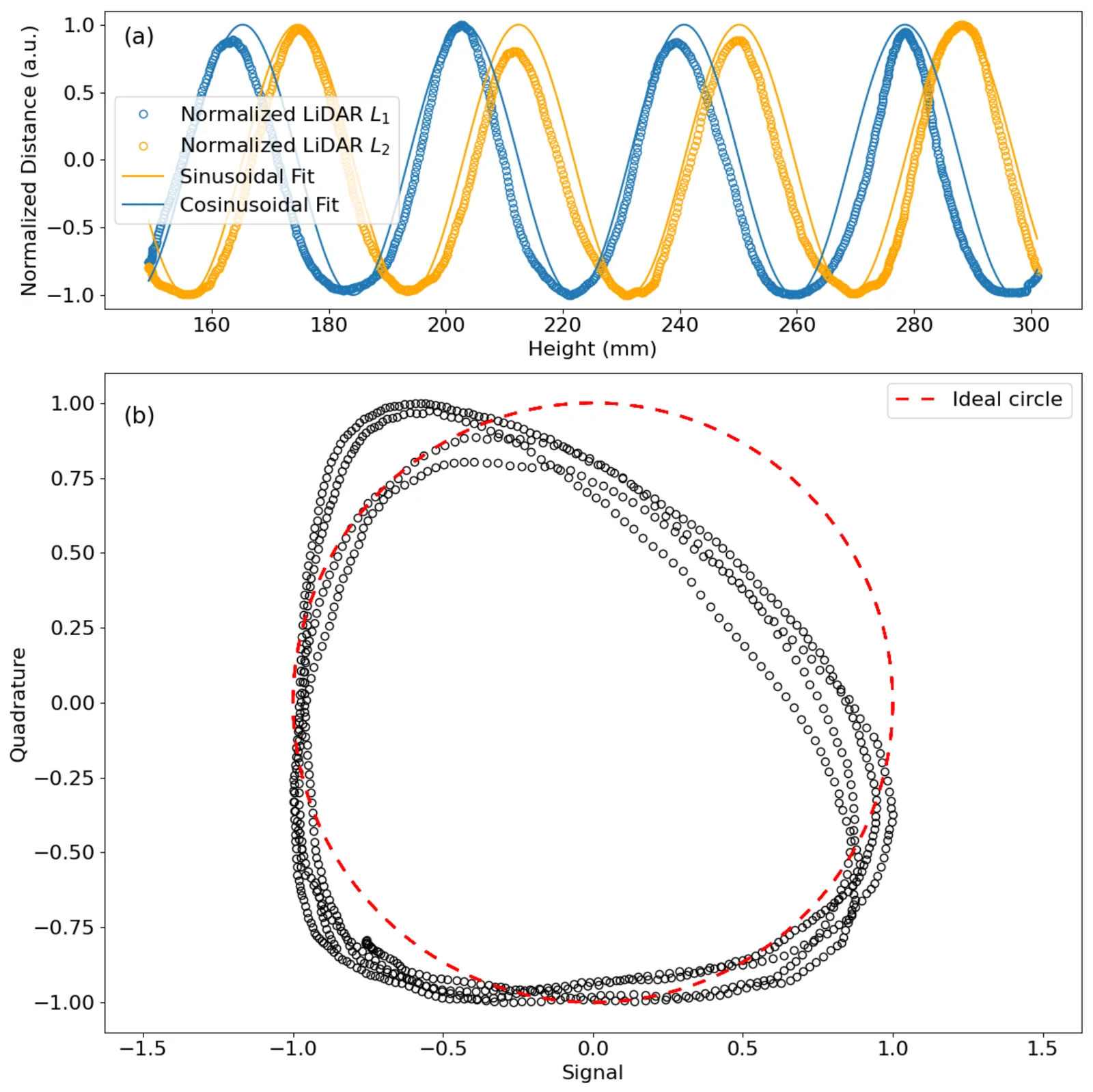
Scan profile with LiDARs $L_{1}$ and $L_{2}$ as a function of height, as measured by $L_{3}$; ideal sinusoidal (and quadrature) fits are also shown (**a**). I–Q plot of the experimental data and the ideal circle from sinusoidal fits. Results for the case of a 35 mm LiDAR-to-spring-coil distance (**b**).

To validate the response characteristics predicted in the previous section, measurements were acquired for the three sensor-to-target distance configurations by averaging 50 samples. During each acquisition, the devices were manually displaced in the vertical direction at an approximately constant speed, sufficiently slow to allow the averaging process to be completed while ensuring continuous data acquisition. The resulting plots are shown in Figure 6, where the in-phase ($L_{1}$) and quadrature ($L_{2}$) LiDAR signals are normalized according to Equation (8). As shown in Figure 6a, the scan profiles of $L_{1}$ and $L_{2}$ as a function of height measured by $L_{3}$ exhibit periodic behavior.

The sinusoidal and cosinusoidal fits are also shown for comparison. In Figure 6b, the I–Q plot shows the expected behavior similar to the previous analysis considering the single-LiDAR characterization for the minimum sensor-to-target distance of 35 mm.

(8) $$\begin{array}{cc} \hat{x}\left(h_{i}\right) & =\frac{x\left(h_{i}\right)-\left(x_{\max}+x_{\min}\right)/2}{(x_{\max}-x_{\min})/2}\\ \hat{y}\left(h_{i}\right) & =\frac{y\left(h_{i}\right)-\left(y_{\max}+y_{\min}\right)/2}{(y_{\max}-y_{\min})/2} \end{array}$$

The phase is obtained directly from the normalized $L_{1}$ and $L_{2}$ measurements using Equation (9):

(9) $$\theta =\mathrm{atan}2(\hat{y}\left(h_{i}\right),\hat{x}\left(h_{i}\right))$$

**Figure 7:**
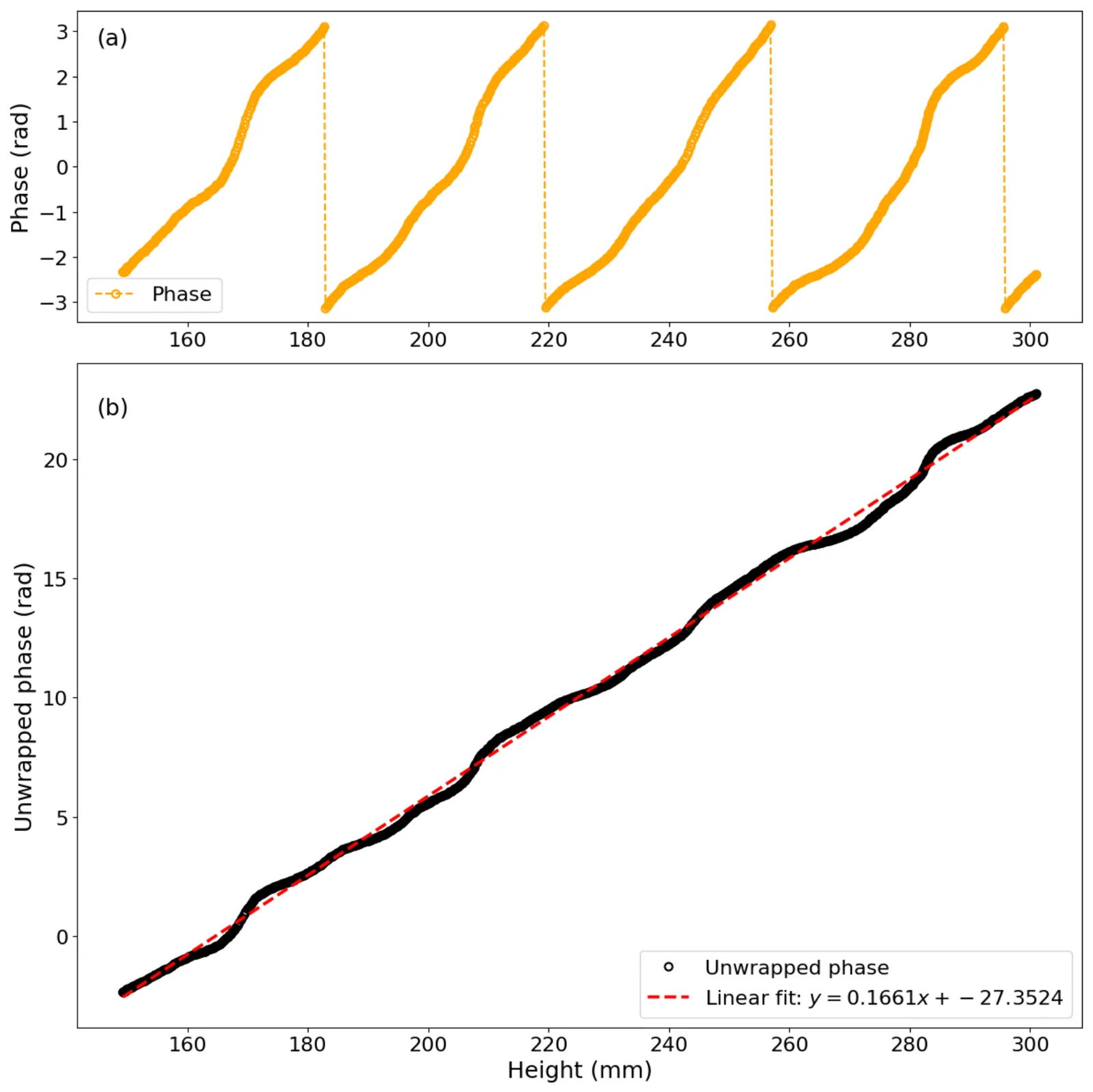
Recovered phase from experimental points as a function of height in millimeters: (**a**) applying the atan2 function; (**b**) the unwrapped phase exhibits linear behavior.

Figure 7a shows the recovered phase from the experimental points as a function of height, *h*, in millimeters, obtained by applying Equation (9). The unwrapped phase is shown in Figure 7b and compared with a linear fit. The behavior is similar to that predicted in the previous section. The height range is different because $L_{3}$ was placed at a different initial height.

**Figure 8:**
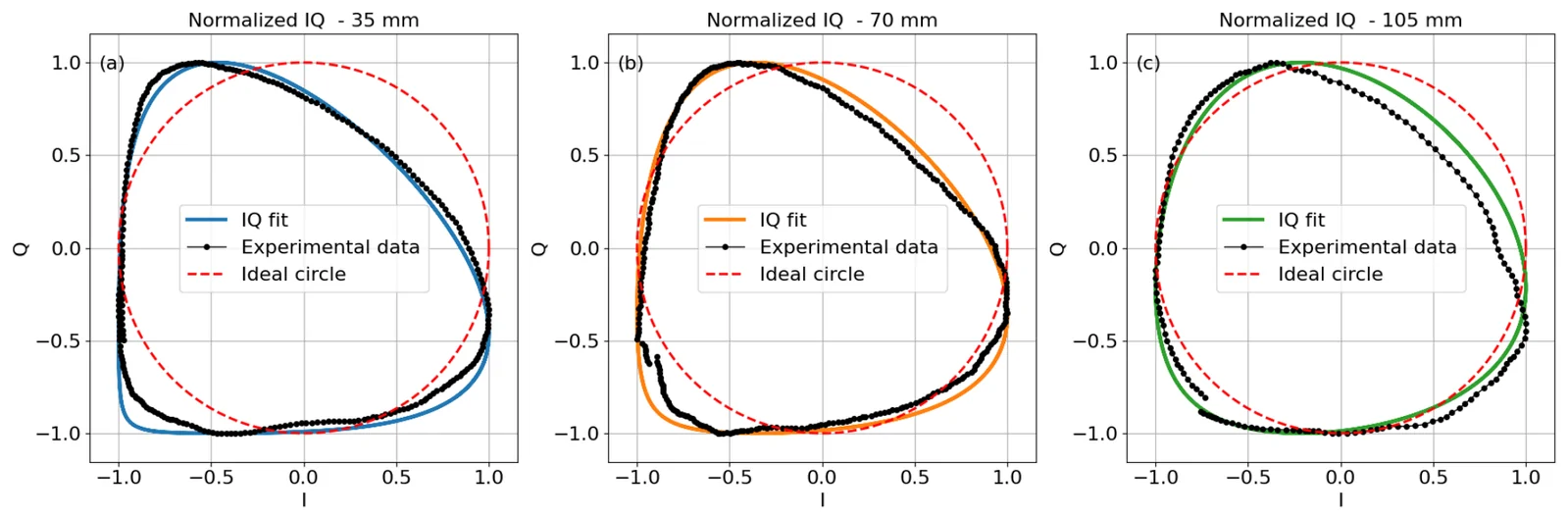
Experimental I–Q plots using $L_{1}$, $L_{2}$, and $L_{3}$ as a function of distance: (**a**) 35 mm, (**b**) 70 mm and (**c**) 105 mm.

The I–Q plots also reproduce the behavior predicted in the previous section, as shown in Figure 8. Since the height and the normalized horizontal distances were acquired continuously by $L_{3}$ and by $L_{1}$ and $L_{2}$, respectively, small differences with respect to the fitted model are expected. In addition, because each measurement corresponds to the average of the previous 50 samples, the experimental results are influenced by the vertical displacement speed during data acquisition. Nevertheless, the RMSE values between the experimental data and the fitted curves remain lower than the RMSE values relative to the ideal unit circle, as reported in Table 4.

The quasi-linear behavior of the unwrapped phase as a function of height can be exploited to develop a tracking algorithm for recovering vertical displacement. While this methodology is applicable to all investigated target distances, shorter distances require a linear-plus-harmonic correction model to accurately convert the measured horizontal distances into height estimates, as illustrated by the periodic response in Figure 5a. This behavior originates from deviations of the I–Q trajectories from an ideal circle. Nevertheless, to reduce computational complexity and facilitate real-time processing, the 105 mm configuration was selected for the tracking experiments. This decision is supported by the quantitative results presented in Table 3 and by the qualitative observations from Figure 4c and Figure 5, which indicate that the 105 mm case exhibits the closest approximation to the ideal I–Q trajectory and the most linear phase response, making it the most suitable configuration for real-time height tracking. The tracking algorithm is described in Section “Height-Tracking Algorithm”.

#### Height-Tracking Algorithm

The height-tracking algorithm is based on the measurements acquired by the two sensors, $L_{1}$ and $L_{2}$. By exploiting the phase relationship between the responses of these sensors, it is possible to estimate the vertical displacement of the bogie spring from horizontal distance measurements. As discussed in the previous section, the phase reconstruction is more accurate when the devices are positioned 105 mm from the target, since the resulting I–Q trajectory more closely approximates an ideal unit circle.

The proposed method relies on the relative phase between the normalized outputs of devices $L_{1}$ and $L_{2}$. The phase is first computed using the atan2 function. Since the phase returned by atan2 is inherently wrapped within the interval (−π,π], a phase-unwrapping procedure is required to obtain a continuous representation of the motion, as previously mentioned. Whenever the phase crosses a discontinuity, its value is incremented or decremented by 2π, depending on the direction of motion. The actual steps are shown in Algorithm 1.
**Algorithm** **1** Height-tracking algorithm: phase-unwrapping procedure Acquire measurements from LiDARs $L_{1}$ and $L_{2}$. Normalize the measurements using Equation (8). Compute θ using Equation (9). Compute $\delta =\theta -\theta_{\mathrm{previous}}$. If δ>π, set δ=δ−2π. If δ<−π, set δ=δ+2π. Update $\theta_{\mathrm{unwrap}}=\theta_{\mathrm{unwrap}}+\delta$. Set $\theta_{\mathrm{previous}}=\theta$. Estimate the height using the fitted $\theta_{\mathrm{unwrap}}$-versus-height calibration curve.

**Figure 9:**
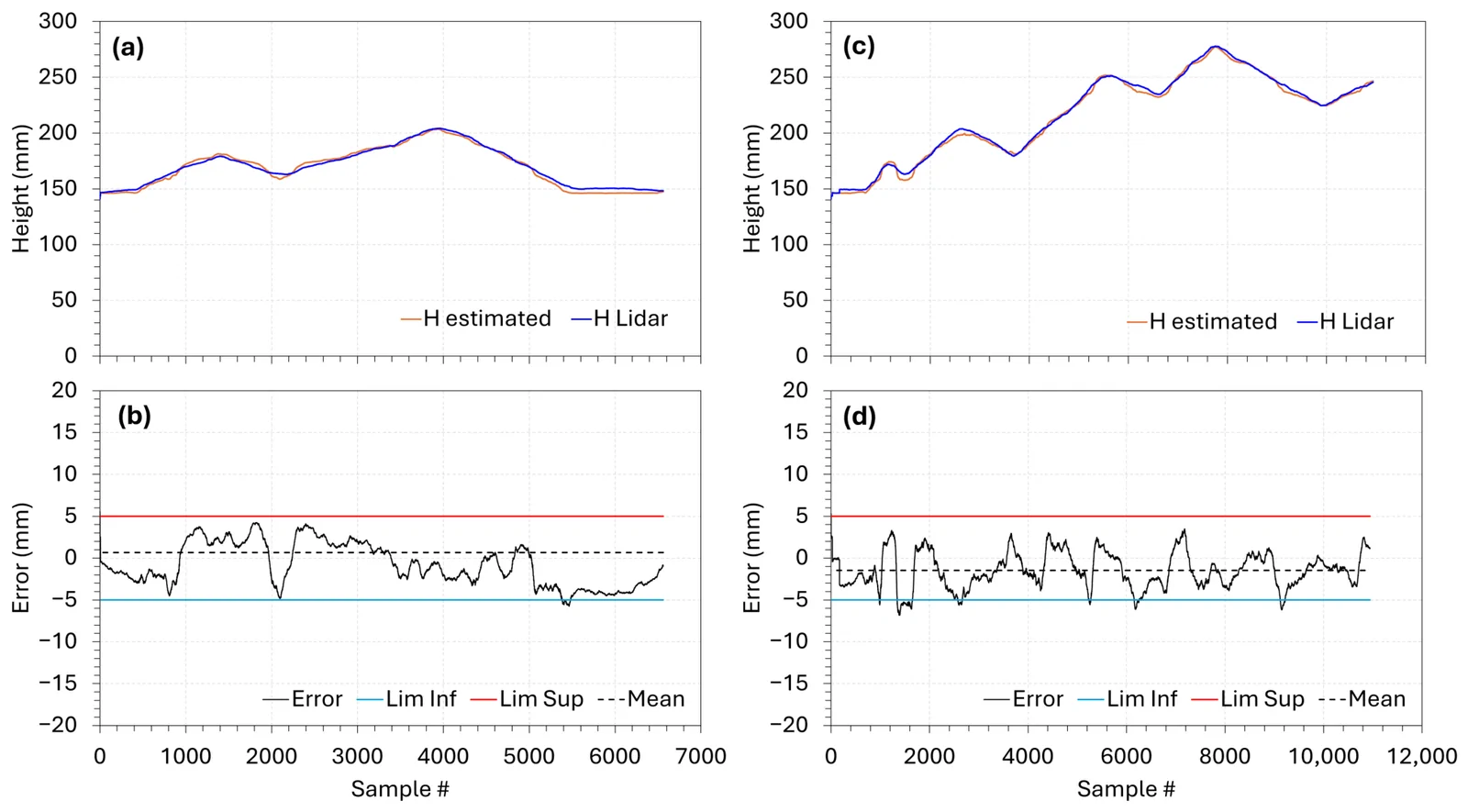
Results of the height-tracking algorithm implemented on Arduino. First vertical displacement test: (**a**) estimated height compared with LiDAR measurement; (**b**) height estimation error. Second vertical displacement test: (**c**) estimated height compared with LiDAR measurement; (**d**) height estimation error.

Figure 9 presents the real-time performance of the proposed height-tracking algorithm implemented on an Arduino microcontroller for the 105 mm sensor-to-target distance, which was selected because it provides the I–Q trajectory closest to an ideal circle and the most linear unwrapped phase response. The estimated height was obtained from the proposed phase-based algorithm and compared with the reference height measured by a third LiDAR sensor ($L_{3}$).

The results of the first vertical displacement experiment are presented in Figure 9a,b. Figure 9a shows that the estimated height closely follows the reference measurement during both upward and downward movements, while Figure 9b presents the corresponding tracking error. Throughout the experiment, the error remains within approximately ±5 mm, with a mean error of 0.66 mm and a standard deviation of 2.35 mm.

A second experiment was performed to evaluate the repeatability of the proposed method. The estimated and reference heights are compared in Figure 9c, whereas the corresponding error is shown in Figure 9d. Similar performance is observed, with the tracking error also remaining within approximately ±5 mm over the entire displacement range. For this experiment, the mean error was −1.47 mm, while the standard deviation was approximately 2.16 mm, demonstrating good repeatability of the proposed tracking algorithm. Possible sources of error include fitting inaccuracies arising from deviations from the ideal linear phase response and the finite resolution of the LiDAR sensor. In contrast, misalignment is not expected to have a significant impact owing to the relatively large FoV of this particular LiDAR device. Nevertheless, considering that the reference LiDAR sensor has a specified distance resolution of 1 mm, the obtained results demonstrate that the proposed indirect height estimation method provides good accuracy while maintaining a low computational complexity compatible with real-time implementation on a low-cost microcontroller. It is worth mentioning that data from the reference LiDAR, $L_{3}$, were also acquired by averaging 50 samples, and part of the final uncertainty is associated with its nominal resolution. The results also validate the I–Q-based phase reconstruction approach presented in the previous sections, confirming that the nearly circular I–Q trajectory obtained at a sensor-to-target distance of 105 mm enables reliable phase estimation and, consequently, accurate vertical displacement tracking.

The Arduino also transmits the estimated height, the reference height measured by $L_{3}$, the normalized distances *x* and *y*, and the unwrapped phase to a Python-based visualization tool. For improved visualization, the normalized distances are mapped onto sine and cosine components through a simple renormalization procedure, generating the corresponding I–Q representation. Video S1 (Supplementary Material) demonstrates the real-time operation of the proposed system. It shows the responses of LiDARs $L_{1}$, $L_{2}$, and $L_{3}$ during successive upward and downward movements of the sensor assembly, together with the reconstructed height, the reference height, and the corresponding estimation error. Appendix A discusses the influence of signal averaging on the temporal system response.

## 5. Discussion and Conclusions

This work demonstrated a low-cost, non-contact method for indirectly estimating the height of a helical bogie spring using two time-of-flight (ToF) LiDAR sensors arranged in quadrature. By exploiting the periodic geometry of the spring, the method converts horizontal distance measurements into an in-phase and quadrature (I–Q) signal pair, enabling real-time phase-based height reconstruction with a low-cost microcontroller (Arduino Uno). Across two independent displacement tests, the method achieved standard deviations of 2.35 mm and 2.16 mm relative to a reference LiDAR (1 mm nominal resolution), confirming the feasibility of the approach despite the modest resolution and wide FoV of the sensors used. The main limitation of the method is its dependence on a periodic target geometry, a condition naturally satisfied by helical springs but not generalizable to arbitrary structures. A calibration step (sinusoidal fitting of the phase-height relationship) is currently required; this could potentially be simplified by leveraging known spring geometry and rapid endpoint calibration during installation. The relatively wide FoV of the selected LiDAR was shown to be advantageous for signal smoothness, but robustness to sensor misalignment and real operational conditions (vibration, temperature, dust, dynamic loading) has not yet been experimentally validated and should be addressed in future work.

Specifically, under dynamic loading, the sensors do not directly measure the applied load or distinguish between compression and expansion of the spring. Instead, each sensor measures changes in its relative distance to the spring surface, and these variations are used to reconstruct the spring height. Thus, compression or expansion of the spring is reflected in the measured signals and, consequently, in the reconstructed spring height. However, the deformation of the spring also changes its effective pitch and, therefore, the relative phase between the signals acquired by the two sensors. As a result, the ideal quadrature condition may not be strictly maintained during significant spring compression or expansion. This aspect represents an important limitation of the current implementation and requires further investigation under dynamic loading conditions.

Future work will focus on: (i) validating the technique under dynamic loading and real railway operating conditions; (ii) establishing a quantitative relationship between spring compression and applied load, enabling indirect load monitoring; and (iii) assessing sensor robustness to misalignment and environmental disturbances. These developments would support the integration of this technique into condition-based maintenance strategies for railway bogie suspension systems.

## Figures and Tables

**Table 1:** Spring topology.

Designation	Parameters
Outer diameter	114 mm
Inner diameter	66 mm
Pitch between coils	37 mm
Wire diameter	24 mm
Circularity	0.989

**Table 2:** Fitting parameters used to generate the reference and orthogonal components (Equations (3) and (4)) for the three spring distances.

Distance	*A*	*B*	*P*	$\theta_{0}$	*k*
35 mm	68.868	58.620	37.144	−1.260	0.316
70 mm	56.174	94.672	37.470	−1.046	0.265
105 mm	37.944	128.970	38.012	−0.607	0.176

**Table 3:** Circularity and phase-linearity metrics for different sensor-to-target distances.

Distance	RMSE Circle	RMSE Linear Phase
35 mm	0.18	0.35
70 mm	0.14	0.27
105 mm	0.08	0.16

**Table 4:** RMSE of continuously acquired experimental data relative to the fits and the unit circle for different sensor-to-target distances.

Distance	RMSE Fits	RMSE Circle
35 mm	0.04	0.17
70 mm	0.05	0.17
105 mm	0.06	0.12

**Figure A1:**
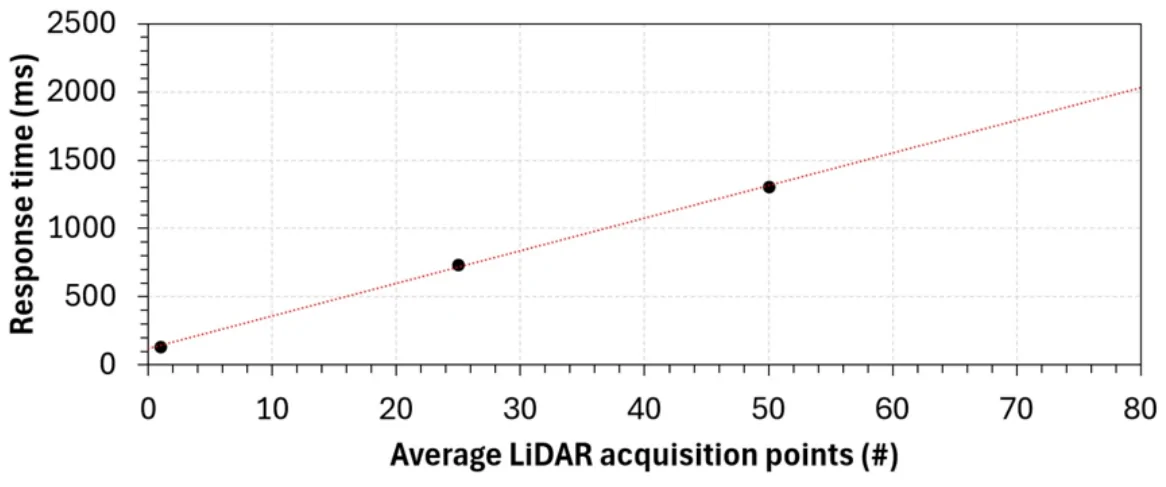
Response time of the LiDAR measurement system as a function of the number of averaged acquisition points.

## Data Availability

The original contributions presented in this study are included in the article/Supplementary Material. Further inquiries can be directed to the corresponding author.

## Supplementary Materials

The following supporting information can be downloaded at https://www.mdpi.com/article/10.3390/s26196183/s1, Video S1: Real-time operation of the proposed technique for indirect height estimation.

## Appendix A

To evaluate the influence of signal averaging on the temporal response of the measurement system, the response time was determined for different numbers of averaged LiDAR acquisition points. As shown in Figure A1, increasing the number of averaged samples results in an approximately linear increase in the response time. The measured response times were approximately 134 ms, 734 ms, and 1304 ms for 1, 25, and 50 averaged acquisition points, respectively. This behavior reflects the expected trade-off between noise reduction through averaging and the temporal response of the measurement system. The dotted line is included only as a guide to the eye.

## References

[1] Fu B., Giossi R.L., Persson R., Stichel S., Bruni S., Goodall R. (2020). Active suspension in railway vehicles: A literature survey. Railw. Eng. Sci..

[2] Ulianov C., Defossez F., Vasić Franklin G., Robinson M., Yannis G., Cohen S. (2016). Overview of Freight Train Derailments in the EU: Causes, Impacts, Prevention and Mitigation Measures. Traffic Safety.

[3] Baykara C. (2025). Investigation of mechanical strength and deformation properties of Y25 bogie suspension systems by finite element analysis. Railw. Sci..

[4] Gorthi S.S., Rastogi P. (2010). Fringe projection techniques: Whither we are?. Opt. Lasers Eng..

[5] Pan B., Qian K., Xie H., Asundi A. (2009). Two-dimensional digital image correlation for in-plane displacement and strain measurement: A review. Meas. Sci. Technol..

[6] Kreis T. (1986). Digital holographic interference-phase measurement using the Fourier-transform method. J. Opt. Soc. Am. A.

[7] Fu Y., Shang Y., Hu W., Li B., Yu Q. (2021). Non-contact optical dynamic measurements at different ranges: A review. Acta Mech. Sin..

[8] Ma J., Zhuo S., Qiu L., Gao Y., Wu Y., Zhong M., Bai R., Sun M., Chiang P.Y. (2025). A review of ToF-based LiDAR. J. Semicond..

[9] Van Der Jeught S., Dirckx J.J. (2016). Real-time structured light profilometry: A review. Opt. Lasers Eng..

[10] Seidaliyeva U., Ilipbayeva L., Utebayeva D., Smailov N., Matson E.T., Tashtay Y., Turumbetov M., Sabibolda A. (2025). LiDAR Technology for UAV Detection: From Fundamentals and Operational Principles to Advanced Detection and Classification Techniques. Sensors.

[11] Zhang S. (2018). High-speed 3D shape measurement with structured light methods: A review. Opt. Lasers Eng..

[12] Su X., Chen W. (2001). Fourier transform profilometry. Opt. Lasers Eng..

[13] Zuo C., Feng S., Huang L., Tao T., Yin W., Chen Q. (2018). Phase shifting algorithms for fringe projection profilometry: A review. Opt. Lasers Eng..

[14] Ryu W.J., Kang Y.J., Baik S.H., Kang S.J. (2008). A study on the 3-D measurement by using digital projection moiré method. Optik.

[15] Li C., Cao Y., Chen C., Wan Y., Fu G., Wang Y. (2017). Computer-generated Moiré profilometry. Opt. Express.

[16] Li C., Cao Y., Wan Y., Li H., Xu C., Zhang H. (2021). Computer-generated frequency-carrier moiré profilometry. Opt. Commun..

[17] Su L., Su X., Li W., Xiang L. (1999). Application of modulation measurement profilometry to objects with surface holes. Appl. Opt..

[18] Lu M., Su X., Cao Y., You Z., Zhong M. (2016). Modulation measuring profilometry with cross grating projection and single shot for dynamic 3D shape measurement. Opt. Lasers Eng..

[19] Wei Z., Cao Y., Wu H., Xu C., Ruan G., Wu F., Li C. (2024). Dynamic phase-differencing profilometry with number-theoretical phase unwrapping and interleaved projection. Opt. Express.

[20] Zheng S., Cao Y. (2013). Fringe-projection profilometry based on two-dimensional empirical mode decomposition. Appl. Opt..

[21] Zhang Y., Cao Y., Wu H., An H., Huang X. (2021). Real-time profilometry by bicolor grating video projection. PLoS ONE.

[22] Tan J., Niu H., Su W., He Z. (2025). Structured light 3D shape measurement for translucent media base on deep Bayesian inference. Opt. Laser Technol..

[23] Qin J., Jiang Y., Cao Y., Wu H. (2025). Single-shot phase-shifting composition fringe projection profilometry by multi-attention fringe restoration network. Neurocomputing.

[24] Urda P., Muñoz S., Aceituno J.F., Escalona J.L. (2020). Wheel-rail contact force measurement using strain gauges and distance lasers on a scaled railway vehicle. Mech. Syst. Signal Process..

[25] Sha Z., Shi X., Gong Y., Qin Y., Qi J., Wang Z. (2026). Measurement method for bogie steel spring parameters based on 3D structured light principle. Eng. Res. Express.

[26] Aela P., Chi H.L., Fares A., Zayed T., Kim M. (2024). UAV-based studies in railway infrastructure monitoring. Autom. Constr..

[27] Dias S., Sousa P.J.S.C.P., Nunes J., Afonso F., Viriato N., Tavares P.J., Moreira P.M.G.P. (2025). Deterministic Light Detection and Ranging (LiDAR)-Based Obstacle Detection in Railways Using Data Fusion. Appl. Sci..

[28] Wu H., Yao Z., Li C., Li J., Lin Q. (2025). Research on Optical Fiber-Integrated Sensor System for Bogie Condition Monitoring of High-Speed Trains. IEEE Trans. Instrum. Meas..

[29] Yoon H.J., Kim J.S., Song K.Y., Cho H.W., Jung J.Y. (2018). Distributed Strain Monitoring of Railway Composite Bogies Using a Brillouin Optical Correlation Domain Analysis System. Appl. Sci..

